# Is There an Ideal REDD+ Program? An Analysis of Policy Trade-Offs at the Local Level

**DOI:** 10.1371/journal.pone.0052478

**Published:** 2012-12-26

**Authors:** George A. Dyer, Robin Matthews, Patrick Meyfroidt

**Affiliations:** 1 The James Hutton Institute, Craigiebuckler, Aberdeen, United Kingdom; 2 F.R.S.-FNRS and Université Catholique de Louvain, Earth and Life Institute, Georges Lemaitre Center for Earth and Climate, Louvain-la-Neuve, Belgium; Cinvestav-Merida, Mexico

## Abstract

We use economy-wide simulation methods to analyze the outcome of a simple REDD+ program in a mixed subsistence/commercial-agriculture economy. Alternative scenarios help trace REDD+’s causal chain, revealing how trade-offs between the program’s public and private costs and benefits determine its effectiveness, efficiency and equity (the 3Es). Scenarios reveal a complex relationship between the 3Es not evident in more aggregate analyses. Setting aside land as a carbon sink always influences the productivity of agriculture and its supply of non-market goods and services; but the overall returns to land and labor–which ultimately determine the opportunity cost of enrollment, the price of carbon and the distribution of gains and losses–depend on local conditions. In the study area, market-oriented landowners could enroll 30% of local land into a cost-effective program, but local subsistence demands would raise their opportunity costs as REDD+ unfurls, increasing the marginal cost of carbon. A combination of rent and wage changes would create net costs for most private stakeholders, including program participants. Increasing carbon prices undermines the program’s efficiency without solving its inequities; expanding the program reduces inefficiencies but increases private costs with only minor improvements in equity. A program that prevents job losses could be the best option, but its efficiency compared to direct compensation could depend on program scale. Overall, neither the cost nor the 3Es of alternative REDD+ programs can be assessed without accounting for local demand for subsistence goods and services. In the context of Mexico’s tropical highlands, a moderate-sized REDD+ program could at best have no net impact on rural households. REDD+ mechanisms should avoid general formulas by giving local authorities the necessary flexibility to address the trade-offs involved. National programs themselves should remain flexible enough to adjust for spatially and temporally changing contexts.

## Introduction

International efforts to compensate developing countries for reducing carbon emissions from forests, an initiative known as Reducing Emissions from Deforestation and Forest Degradation (REDD+), have focused most recently on developing implementation mechanisms at the national and sub-national levels [1], [2]. A key challenge is how to reduce emissions without disadvantaging vulnerable sectors of society. Public benefits accruing from reducing emissions have to be set against social justice questions such as who will be the private winners and losers of REDD+ and how to avoid forcing rural households to abandon their livelihoods [1], [3]–[6]? Indeed, emissions from all land-based economic sectors eventually should be considered in a comprehensive emissions-reduction strategy–it is not a “forest problem” alone. A broad perspective is required due to the interdependencies of international markets for agricultural and forest products, but a local perspective also is necessary [1], [2]. An integral policy must target incentives at the various actors and levels involved in REDD+, from international buyers and sellers of carbon services to the people at the “coal-face” who must change their land-use practices. Actors potentially include national and sub-national governments, industries and individual businesses, local communities, indigenous peoples, producer organizations and individual farmers as well as those responsible for administrating REDD+ programs and verifying their outcome [1]. Most of them will be required to make REDD+ work, but they will also expect a share of the benefits, which could compromise REDD+’s effectiveness, efficiency and equity–the 3Es. Appropriation of benefits by some of these actors most likely will result in fewer benefits further along the chain, risking that emission reductions might not be delivered and further flows of funds dry up, so that everyone suffers. While solutions are likely to vary from country to country, without addressing these crucial links between global and local, the climate policy discussions run the risk of divorcing themselves from reality.

In principle, previous experience could provide a basis to design the ideal REDD+ program [2]–[5]. Over the last decade, a wide variety of schemes for reducing deforestation and forest degradation have been tried and tested around the world, including various forms of Payments for Environmental Services (PES) [5]–[8]. Unfortunately, most of these efforts have been characterized by a general absence of monitoring and critical evaluation [3], [7]–[8]. This limitation can be circumvented to some extent through the use of simulation methods [9]–[14]. Simulation methods used in land-use analysis range from the extrapolation of observed patterns [13] to explicit models of agent behavior [9]–[11]. Understanding this behavior is necessary for policy design and analysis [15]–[17]. Policy simulations run the gamut from simple calculations using current data [12] to projections based on dynamic optimization and partial equilibrium models [10]. These models have been used mostly to explain the association of deforestation rates to land rents and carbon prices, but their specific goals can be diverse. Models calibrated on economic, demographic and technological expectations are used to generate short and long-term projections [10]; other models are conceived as stylized frameworks to search for the ideal REDD+ mechanism [11], [14].

In this paper we use general equilibrium simulation methods to analyze the direct and indirect effects of a simple REDD+ program and the trade-offs arising between its 3Es at the village level. We simulate several variations on a basic scenario to assess the extent to which alternative reforms deviate from an ideal Pareto outcome (i.e., an efficient outcome where no one experiences net losses). Simulations are designed to highlight the effect of program design given a particular local context, leaving the role of context to be discussed in a subsequent paper. The particular context of this paper–i.e., a Mexican highland village with a mixed subsistence/commercial agricultural sector–allows us to address a pressing but largely overlooked issue: the potential interaction between REDD+ and food production in vast areas of the developing tropics where subsistence agriculture is the norm. To this end we show how outcomes change after incorporating the value of non-market goods and services provided by subsistence activities. In the remainder of this section and the next we present the theoretical and methodological framework of our work. A subsequent section describes the policy scenario and its expected outcomes. A concluding section considers the options available to policy makers charged with designing REDD+ while reflecting on the robustness and generality of our results.

### A Theory of Change: REDD+’s Causal Chain

Studies modeling REDD+’s causal chain have linked the potential outcome of international negotiations (i.e., the setting of reference levels and/or carbon prices) to land use decisions at a very fine scale in order to develop mechanisms to implement REDD+ across the globe, design incentives for national governments to join or to assess the indirect impacts on global commodity markets [10]–[12], [14]. While the efficiency of a particular mechanism can be secondary, the distribution of costs and benefits across countries is considered the basis of its political feasibility and hence of its effectiveness [12]. Most of these studies have assumed that governments would first commit to specific reductions in deforestation at the national level and later develop policies to achieve these commitments. Not surprisingly, REDD+’s causal chain at the sub-national level is extremely simplified in these analyses.

A recent study addressing the sub-national level considers the potential distribution of costs and benefits between national and district governments [14]; but to our knowledge, no study has considered the diversity of actors at the local level and the causal processes of which they are part. It is these actors who will be directly responsible for land-use decisions, and their potential gains and losses will determine REDD+s acceptability at this level [5], [18]–[19].

**Figure 1:**
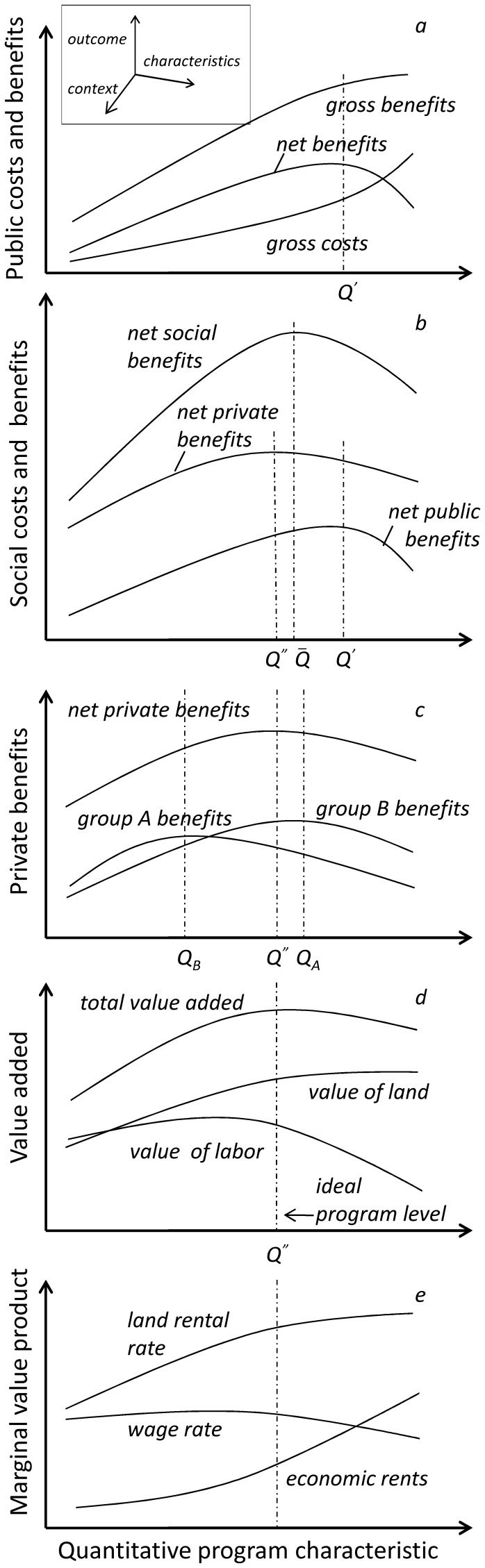
REDD+’s causal chain. REDD+’s public costs and benefits can be conceived as a function of specific program characteristics, whose influence can be traced from their effect on rents and wages to the associated distribution of gains and losses across different sectors of the population.

A common assumption of most previous studies has been that the decisions of national planners and landowners/users coincide. That is, that the social benefits of REDD+ are greatest when the net revenue derived from the land is maximized. This is not necessarily the case. Designing the ideal REDD+ program would require anticipating the full distribution of gains and losses that might follow implementation. In theory, given sufficient data, it should be possible to graph REDD+’s public costs and benefits as a function of specific program characteristics, as we do hypothetically in Figure 1a. Such a graph would reveal the level at which the gross costs and benefits are furthest apart (*Q’*) as the point where the net public benefits of REDD+ are highest. Clearly, both axes in this figure represent single dimensions of a more complex policy problem set within a heterogeneous socio-economic, political and environmental context [4], [17]. That is, the policy outcome should be construed as a dimension mapping onto the independent, multidimensional space of program characteristics and context variables (see inset). Anticipating the outcome of alternative combinations of characteristics given a particular context is then the prerequisite to identifying the program that maximizes public benefits in a specific locality. This program will also generate private gains and losses for various sectors of society, including local landowners, who may see the program as less than ideal.

Accommodating private interests may require attending to program characteristics not considered up to this point, e.g., the equity of its distribution of payments. Characteristics with both public and private implications could create trade-offs, forcing authorities to settle for a compromise (Q*) solution–one that falls short of public goals but maximizes social (i.e., public+private) benefits, as shown in Figure 1b. The heterogeneity of private interests could hinder this solution, for REDD+ could have distinct impacts on different groups of stakeholders. For instance, the costs and benefits experienced by program participants should differ from those of non-participants (Fig. 1c). Cost-benefit analysis could help anticipate the distribution of gains and losses for different stakeholder groups–an increasingly common requirement of government regulation [20]; but implementing a feasible program could depend on skillful negotiation more than on benefit maximization [8]. Economic analyses can contribute to the political process nevertheless by unraveling the potentially complex association of outcomes with program characteristics and local contexts–i.e., REDD+’s causal chain–which so far we have treated in Figure 1 as a black box.

While we referred above to program characteristics as independent variables, this might not always be the case. Depending on the choice of instrument, causal relationships might arise between particular program characteristics, thus restricting the options (i.e., the combinations of characteristics) available to program authorities. This is clearly the case of incentive-based mechanisms such as Payments for Environmental Services (PES), which give flexibility to potential participants rather than to authorities [4], [8]. In a PES program, for instance, authorities must offer competitive prices for environmental services (e.g., carbon management) to reach a given enrollment target–or they may retain control over prices but not on how much land is enrolled. Although not always an option, command-and-control mechanisms, by contrast, would allow authorities to determine both the area to be enrolled and the kind of compensation paid to landowners.

So far we have also referred to REDD+’s outcome as a one-dimensional variable–i.e., monetary costs and benefits–determined by exogenous drivers, but this too need not be the case. Official estimates of the “social cost of carbon” often reduce to a single monetary value [20]. Yet, there are additional ways of assessing social outcomes, e.g., in terms of the 3Es [4], [21]. Acknowledging the interrelation of outcomes along different dimensions also is important. It is widely known, for instance, that the price paid for environmental services has simultaneous implications on the equity and efficiency of PES [4], [7], [8]. But the implicit trade-off is not only a function of exogenous factors (e.g., land characteristics and market prices) since equity and efficiency also depend on the program’s (endogenous) spillover (or economy-wide) effects [12]. PES’s effectiveness also can be compromised by this spillover. The indirect effects of PES on the rural economy, for instance, can influence participants’ willingness to remain in the program and non-participants willingness to join, and other land-use decisions. Yet, any adjustment that restores the program’s effectiveness will simultaneously alter the trade-off between efficiency and equity. By and large, addressing these trade-offs or taking advantage of the potential synergies in REDD+ requires understanding the causal mechanisms involved [5], [8]–i.e., identifying the processes linking the axes in Figure 1b–and these mechanisms often involve economy-wide processes.

Carbon-management programs could have multiple effects on the value and productivity of rural assets, which is the crucial link in REDD+’s causal chain. In the long run, REDD+’s effects could depend on whether interventions create incentives for investment and innovation [4]. In the short term, which is the focus of our work, interventions could influence the marginal productivity of land and labor by determining how intensely these factors are used. Most effects will depend at once on the nature or design of the program put in place and the local context [16], [17]. Since markets require that each factor’s returns (i.e., its marginal value product, which is equal to its marginal productivity times the value of its output) equal its price, any characteristic that impacts the demand for these factors could influence their market value simultaneously. A program’s spatial scale or the price paid for services may have distinct, quantifiable effects on the rents received by landholders or the wages paid to local workers (Fig. 1e). The same characteristics could also determine the creation of economic rents–i.e., the portion of payments in excess of the opportunity cost of land–available only to participants. Whether these effects are positive, negative or nil will depend on the structure of local markets [16]. A small land set-aside program might have few effects in an economy with large and complete markets. But the same program could have a significant influence on land-use choices wherever markets are imperfect, small, closed or absent altogether, as often is the case in the developing world. Evidently, the outcome will differ across localities, perhaps markedly, but it might also differ across farms within each locality. The reason is that households in developing areas often experience varying degrees of access to markets and other limitations (e.g., subsistence or liquidity constraints) that tie their production decisions–and thus the productivity and value of their assets–to their income, including potential PES transfers [22]–[23].

Mapping the effects of specific program characteristics on productive assets (Fig. 1e) places us a step away from identifying a program’s social outcome within a particular context (Fig. 1c). The causal chain is fully mapped after wages, land rents and economic rents are translated into estimates of the value added accrued or paid by different economic actors–i.e., the costs and benefits experienced by various stakeholders (Fig. 1d). The complete mapping of the causal chain in Figure 1 thus constitutes the theory of change that is essential to impact evaluation, and which also brings us a step closer to designing a place-based REDD+ policy [5].

**Figure 2:**
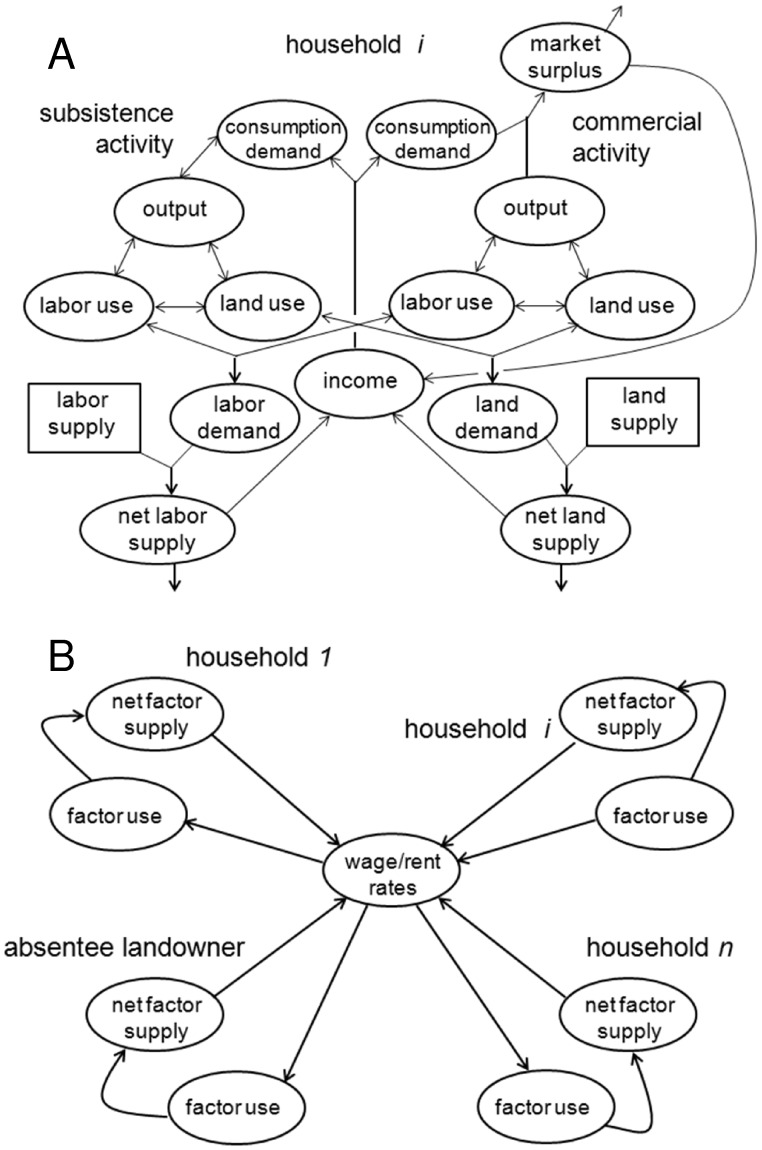
The village model. The model consists of 49 households engaging in various on-farm activities (A) while interacting off-farm with other agents in the village economy (B). Ovals represent variables (i.e., agents’ decisions or endogenous factor prices); rectangles represent fixed endowments; arrows indicate causality. A) Output and factor inputs are determined simultaneously for each activity. When aggregated across activities, factor use constitutes the household’s factor demand. The difference between factor demand and supply determines its net factor supply to the market. B) When aggregated across households, net factor supplies determine factor prices within the village, which influence each household’s factor use simultaneously.

Other authors have relied on analytical methods to decipher the linkages described in Figure 1 [16], but such models are necessarily limited in scope. A model representing the complexity of social systems (including the heterogeneity of actors involved and their interactions) is not likely to have an analytical solution. Figure 2 represents such a model, where each household makes numerous simultaneous decisions based on prevailing market conditions, including REDD+ policy (Panel A). However, when aggregated across the population, households’ decisions influence these same conditions, feeding back into their decision-making process (Panel B). It is this complex process that lies behind the potential association of program characteristics with wage and rental rates (Fig. 1e).

While not a substitute for analytical approaches, simulation methods are much better equipped to handle this complexity. Agent-based models, in particular, can help unravel the potential intricacies of REDD+ policy, which has been characterized as a multi-level PES scheme for carbon management [21], [24]. We use an agent-based model in a general equilibrium context to explore the implications of a simple PES program, retracing the causal chain described in Figure 1 to reveal how various processes interlock to produce a particular outcome in a specific context. Simulation results then are used to generate empirical versions of Figure 1, thereby mapping the changes introduced by the program’s reform into this chain. More specifically, results describe how various program characteristics are linked to changes in factor productivity and how these changes are tied in turn to the wellbeing of various stakeholders.

## Methods

### An Agent-based, General Equilibrium Model

Our methodological framework integrates multiple household models into a single model of a local economy in a developing area. The model (described in detail in Supporting Information S1) is based on the village of Zoatecpan, a farming community in densely-populated central Mexico, and calibrated using survey data for a sample of households in this locality [9]. Each household in the sample–i.e., 49 households representing 10% of the population–is modeled as an independent decision maker that engages in on- and off-farm activities (Fig. 2A) and interacts with other agents via markets (Fig. 2b). The economy thus consists of the activities and interactions of multiple non-exclusive types of agents (Table 1). The system is further defined by closure rules that describe markets operating in the locality. When land, labor and maize markets are defined as strictly local, the locality can be described as a closed system where there is little room for the displacement (or leakage) of carbon emissions out [15]. When markets extend beyond village boundaries, the system is open and leakage is likely. In a closed system, the demand and supply of each good, factor and service is satisfied through local adjustments in prices (i.e., price equilibrium); in an open system, wages, rents and prices are determined in the larger economy and considered fixed. In both cases subsistence activities are influenced by their own, implicit value (or shadow price), which is the sum of the market and non-market value of the goods and services they provide.

**Table 1:** Types of agents (as defined by their assets and activities).

Ownership of land	Landholders (94% of households and absentee landowners)	Landless households (6% of households)
Cultivation of maize	Maize farmers (98% of households)	Non-farmers (2% of households)
Sale/purchase of maize	Commercial farmers (4% of households) sell maize surpluses, consume a fraction	Subsistence farmers (94% of households) and landless households buy maize
Land rental	Landlords (2% of households and absentee landowners)	Tenants (2/3 of landless households and 35% of landowners)
Labor hire	Employers (48% of households and absentee landowners)	Employees (48% of households)
Program participation	Participants (variable number of households and absentee landowners)	Non-participants (variable number of households)

We consider a partially open system where wages and rents are determined in local markets but maize prices are those of the larger economy; i.e., households can trade maize outside the locality but not land or labor–at least in the short term. Alternative closure rules are used in a second scenario to analyze the sensitivity of results to these conditions. Assumptions on price formation can be used simultaneously to model the program’s scope; i.e., depending on these assumptions the village can represent either the entire area in PES or only part of its area of coverage. Our closure rules are compatible with the assumption that the locality is one of many contiguous localities involved in PES.

All simulations involve a simple policy program that requires landowners to set aside arable land for forest recovery. A reforestation scheme of this sort would be an option under REDD+ if reference levels were based on total forest cover change, i.e., on net rather than gross deforestation, and could generate substantial co-benefits [24]–[26]. It also allows us to leave aside momentarily the problem of defining a reference level for deforestation and the ensuing need to forecast complex system dynamics [14], [27].

Scenario 1 considers the introduction of the basic program. Scenarios 1a and 1b (and 1c in Supporting Information) introduce alternative reforms that administrators might implement *ex post* to address specific shortcomings. These scenarios reflect the real-life constraints of reforming an existing program. Finally, scenario 2 reconsiders the program’s introduction in a slightly different context. Since most results are independent of scale, we report percentage changes but refer occasionally to absolute figures to provide a reference point. These figures are based on detailed survey data, but they should not be construed as indicative of actual opportunity costs and carbon prices needed in the study area, which would require measurement of biophysical variables beyond the scope of the present work. In order to restrict the number of possibilities considered here, we make a number of simplifying assumptions, focusing on landowners’ willingness to participate in PES without considering their eligibility or ability to participate. The only rule is that local landowners are given priority over absentee landowners.

Simulations assume no eligibility requirements other than to set aside any amount of land; i.e., participants can pool resources to avoid minimum-area requirements and reduce fixed costs that may otherwise constrain small holders’ participation [6], [28], [29]. As in previous carbon-sequestration programs in Mexico, participation requires no long-term commitment, allowing us to focus on its short-run implications. Extraction of lumber and other forest products is not allowed, which implies that potential benefits associated with an expanding supply are not assessed. Marginal environmental benefits are assumed to be constant; i.e., they do not change with the scale of implementation, which is reasonable in the case of carbon sequestration. Finally, soil quality, microclimate and other biophysical attributes of the land are assumed to be homogenous across the landscape. Thus, intrinsic agricultural yields as well as total biomass densities are fixed and uniform, which implies that both land rental rates and environmental benefits per unit area also are uniform. This last assumption is somewhat restrictive since the marginal cost curve of carbon services (and their social repercussions) could depend on the heterogeneity of land and its distribution [10]–[12], [16]. A more complex set of incentives might be necessary to achieve effective and equitable outcomes when the marginal environmental benefits are not uniform [4]. Nevertheless, our assumption allows us to keep this source of variation neutral in order to identify other factors with similar effects.

### Scenario Results

#### Scenario 1. A cost-effective program

The first scenario considers the introduction of a program that seeks to achieve its goal at a minimum cost to the public. The goal is to sequester an amount of carbon equivalent to allowing forest cover to return to 10% of arable land in the locality. Program administrators set carbon prices by calculating the equivalent per-area payment that will secure the level of enrollment desired–i.e., landholders’ “willingness to accept” a payment in exchange for forfeiting the right to use the land [30]. In our model economy, this requires offering participants a payment 7% greater than what they would receive in the land market, i.e., 7% above the original rental rate (Table 2, column a). At this price, carbon management is clearly an enticing alternative to renting land out to farmers, and so all local landowners who previously rented land out–i.e., landlords–now devote this land to PES. This group represents only 2% of households in the locality but owns 13% of the land. Other landowners are constrained to some degree by their own subsistence demands; still, 40% of local households enroll part of their landholdings into PES. Since the total land area in the locality is fixed (i.e., supply is inelastic), market rents rise until demand meets supply (i.e., market equilibrium is restored). That is, by design, rents rise by 7%, raising the opportunity cost of enrollment to the level where landowners are indifferent between alternative land uses. At this point, local landowners contribute 97% of the program’s target (Table 2), but absentee landholders supply the remainder, which represents a small proportion of their landholdings.

**Table 2:** Economic effects of alternative PES program designs (as defined by program characteristics).^1^

	Scenarios						
	1 (1a, 1c)	1a	1b		2		1c
*Program characteristic*s	(a)	(b)	(c)	(d)	(e)	(f)	(g)
Carbon prices^2^	7	50	14	14	3.7	17	15
Program area^3^	10	10	10	20	10	20	20
*Participation and enrollment*							
Participating households^4^	40	40	40	52	21	52	52
Local enrollment of land^5^	97	96	97	62	84	59	62
*Factor prices*							
Wages	−2.5	−2.6	−2.5	−5.3	0	0	−5.3
Land rents	7.0	7.1	7.0	15	3.7	17	15
Economic rents^6^	0.0	40	6.6	−1.0	0	0	0
*Crop output*							
Total output	−3.2	−3.2	−3.2	−6.6	−8.3	−10	−6.6
Subsistence-farm output	0.3	0.8	0.4	0.7	−0.3	−1.1	0.8
Commercial-farm output	−12	−13	−12	−25	−28	−32	−25
Market surplus	−22	−24	−22	−46	−52	−57	−46
Open-market purchases	2.9	3.4	2.9	6.0	9.3	10	6
*Incomes (in real terms)*							
All households	−0.8	0.6	−0.6	−1.7	0.0	0.1	−1.6
Subsistence farmers	−0.8	0.7	−0.5	−1.6	0.0	0.1	−1.6
Commercial farmers	−1.0	−0.9	−1.0	−2.1	0.0	0.2	−2.1
Program participants	−0.8	1.9	−0.3	−1.7	0.1	0.2	−1.6
Non-participants	−0.8	−0.7	−0.8	−1.6	0.0	−0.1	−1.6
Local landlords	−0.1	29	4.7	−1.0	0.5	2.0	−0.1
Absentee landlords	6.9	7.6	7.0	15	3.7	17	15

1. Figures represent percentage changes with respect to the baseline before the program, except as noted below. Columns represent start and end-points of a scenario, but some points are common to several scenarios.

2. Percentage in excess of original rental rates.

3. Percentage of total arable land in locality.

4. Percentage of total village households.

5. Percentage of program target.

6. Percentage in excess of current rental rates.

Setting land aside has economy-wide repercussions. Agricultural output declines by 3.2%, and given that the program does not hire workers that are laid-off in the process, local wages drop by 2.5% (restoring labor-market equilibrium) (Table 2). The average household is a net supplier of labor but also rents land from a landlord. Thus, changes in wage and rental rates represent an unfavorable shock to its terms of trade (i.e., the quantity of land it can rent with a day’s wage decreases), reducing aggregate nominal income in the locality by 1.0%. Subsistence agriculture dampens this shock slightly because lower wages allow farmers to use more labor to intensify food production. Consumption demand for subsistence goods and services diminishes simultaneously due to housholds’ loss of purchasing power, decreasing the implicit value (or shadow price) of subsistence output. Accordingly, average incomes decrease only 0.8% in real terms. Overall, the resilience of subsistence farming forces commercial farmers to shoulder the program’s burden and reduce their use of land significantly. Their output and surplus decrease by 12% and 22%, respectively (Table 2). The existing food deficit in the locality grows by 2.9%. This shortfall requires purchasing maize in the open market and hence could contribute to program leakage [15].

**Figure 3:**
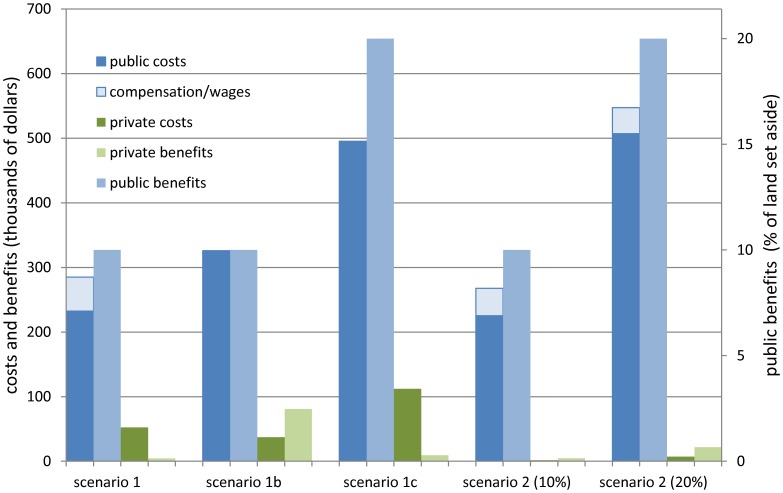
Aggregate costs and benefits of alternative program designs. Alternative program designs, represented through various scenarios, yield vastly different costs and benefits. The net social benefits from REDD+ are given by the sum of its public and private benefits minus the sum of its public and private costs.

Average income losses conceal wide variation among economic agents. Carbon prices momentarily create economic rents equivalent to 7% of rental rates, but these disappear as soon as land rents rise. Thus, *ceteris paribus*, program participants are not better off than non-participants (Table 2). Gains and losses nevertheless are distributed unevenly within these groups based on each household’s factor endowments and market positions: the combination of higher rents and lower wages favors the landed but hurts working families [16]. Not surprisingly, local landlords fare better than their neighbors, but their gains on rents barely offset significant wage-income losses (whether actual or imputed). Absentee landlords do not suffer similar losses, since they do not sell their own labor in local markets. Hence, they are the only group experiencing net gains, which amount to $4,000, a 6.9% gain. Overall, the program transfers $232,500 to participating landowners but entails even greater costs to the community, all of which amounts to a net private loss of $48,200 in real terms. These figures constitute the net public and private costs of carbon services, respectively (Fig. 3).

In sum, the program is effective and efficient by design, but these qualities come at a cost to local households. Although absentee landowners accrue net benefits, redistributing their gains to reduce others agents’ losses–e.g., through taxes–would jeopardize the scheme’s effectiveness. Moreover, only $590 of the $4,000 in private gains comes directly from carbon payments, the rest being collected from local farmers via rent increases. A more equitable outcome could be reached if the program absorbed all private costs, e.g., by compensating losers via unconditional cash transfers in the amount of households’ (real-term) losses. This would entail a 23% increase in the program’s public costs (without considering any administrative costs) (Fig. 3). A more efficient solution might be to redesign the program to internalize its unexpected private costs. Reforms might be obstructed by program rules or the creation of entitlements nevertheless. For instance, authorities might be able to change the price of carbon but not to redistribute existing entitlements. Alternatively, they might be contractually obligated to pay a given price for carbon but at liberty to raise the program’s target. With the first option they would rely on program benefits trickling down from participants to other affected households, while their aim with the second option would be to encourage wider participation to increase the number of households benefiting directly. We explore these two options in scenarios 1a and 1b, respectively.

#### Scenario 1a. Improving equity through higher carbon prices

This scenario simulates a gradual increase in the price of carbon up to 40% above the original price. Such an increase is equivalent to raising per-area payments from 7% up to 50% in excess of the original market rents, which could entice a greater number of landholders to devote larger areas to carbon sequestration. In this scenario, however, the amount of carbon sequestered remains unchanged, so the program’s total area is kept constant and enrollment open to current participants only. (Scenario 1c, in Supporting Information, relaxes these assumptions.) That is, as prices increase, participants in the first scenario must decide whether to leave or remain in the program and how much land to enroll, provided that the total does not exceed the set target.

**Figure 4:**
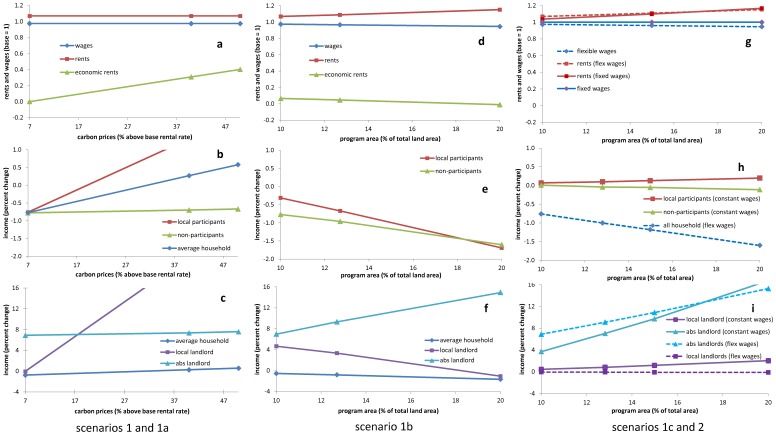
Effects of program design on key economic variables. Changes in carbon prices and land area under a simulated PES program and their effects on value added and economic rents (top row), local households’ income (middle row), and landlords’ incomes (bottom row).

Unsurprisingly, all participants remain in the program, but many reduce their enrollment just slightly as carbon prices rise (Table 2, col. b). The reason is that as their incomes increase, their demands for subsistence goods also grow; i.e. farmers prefer to devote land to farming rather than forest as the implicit value of subsistence output rises. This “income effect” is analogous to the one observed in the classic farm-household model, where income gains associated with rising staple prices result in greater on-farm consumption of staples and thus smaller market surpluses [31]. In our case, subsistence consumption/production expands up to 0.8%, pushing rents up a notch as agricultural land use increases and forcing commercial agriculture to contract. Since productivity is lower in subsistence farms than in their commercial counterparts, aggregate output also declines gradually (Table 2). Market surpluses decrease (up to 24%), and the local grain deficit increases (up to 3.4%). Wages continue decreasing as carbon prices rise, albeit at a very low rate: up to a 2.6% decrease in wages and a 7.1% increase in rents (compared to −2.5 and 7.0% changes, respectively, observed in the first scenario) (Fig. 4a). Compared to the retirement of land, an increase in carbon prices has few economy-wide effects but a noticeable income effect that buffers the program’s impact in general.

As expected by design, program changes have their greatest impact on wellbeing. Average income losses in the locality diminish gradually as carbon prices rise, and they become gains when prices reach a level 33% above market rents (Fig. 4b). At prices 50% above these rents, net private gains in the locality add up to $43,700 in real terms (Fig. 3). The program’s public costs increase accordingly, i.e., up to $325,900 or 14% more than in the first scenario after compensation. Thus, this is less efficient than compensating households directly, and less equitable: 0.8% income losses for the average program participant become 1.9% gains in real terms (Fig. 4c), but many participants and most non-participants–79% of all local households–still experience losses that amount to $37,300 in real terms (Fig. 3). The worsening terms of trade are directly responsible for these losses; but ultimately it is program rules that determine the distribution of gains and losses by creating considerable economic rents but preventing them from spilling over to non-participating landowners (Fig. 4a,b). That is, due to restrictions on enrollment and participation, economic rents are not transformed into higher market rents that would benefit landowners in general (as in scenario 1). Program rules also determine the distribution of benefits among participants, allowing former local landlords to benefit the most (Fig. 4c). Their income increases by 28.6%. Absentee landlords’ income increases by only 7.6% because their gains depend mostly on market rents, which do not increase significantly as carbon prices rise. Local and absentee landlords’ gains amount to $69,800 and $4,400, respectively.

In sum, higher carbon prices are highly inefficient as a form of compensation, reducing individual agents’ losses by only 23% (from $48,200 to $37,300) (Fig. 3). Overall, changes do not improve the program’s equity because payments are not distributed according to individual losses and there is no significant spillover of benefits to non-participating landowners via rents or to other households via an economy-wide multiplier. The multiplier effect of cash injections is negligible because goods markets in the locality are relatively well integrated into the greater economy. Using carbon prices to improve equity has an additional drawback: it increases the program’s public costs without adding to its public benefits, thereby dissipating its net gains (Fig. 3).

As mentioned earlier, another option would be to allow wider participation while generating greater public benefits. There are two possible courses of action for program administrators. If payments per unit area already exceed land rental rates (which entails economic rents), the program’s target can be raised without raising carbon prices. In fact, economic rents can be defined as a payment in excess of what is necessary to secure enrollment, so their existence guarantees an abundant supply of land to the program. On the other hand, if economic rents are nil, increasing enrollment will require increasing carbon prices. We analyze the first option next and report the second option in scenario 1c (see Supporting Information).

#### Scenario 1b. Improving efficiency and equity through wider participation

We now assume that having realized the inefficiency of raising carbon prices, program administrators change course half-way through the previous reform. That is, after raising prices to the equivalent of a payment 14% in excess of market rents while holding the enrollment target constant, they now raise this target gradually (up to 20% of total land in the locality) without further price increases. Since the price of carbon is still enticing–i.e., participants initially collect economic rents equal to 6.5% of current rental rates–the number of participants increases from 40 to 52% of local households by the time the program’s area doubles (Table 2, col. c, d). All new participants are subsistence farmers who reduce the extent of their cropping to engage in carbon services. But local landlords have no additional land to enroll, so the area committed grows only by 27%; i.e., local enrollment cannot keep pace with the program’s expansion. In the end, local landowners contribute only 62% of the target (down from 97%) while absentee landlords supply the remainder, increasing their contribution substantially (i.e., >2,000%).

The economy is more sensitive to changes in the availability of land than to cash flows, so the program’s expansion has a much greater impact than carbon prices had in the previous scenario. As the area enrolled doubles, rents increase by 15% and wages decrease by 5.3% (Fig. 4d). Rent increases result in a significant contraction of agricultural activity. Aggregate output declines 6.6%, but subsistence consumption/production continues unabated, financed by program transfers. Doubling the program’s area has almost the same income effect as doubling carbon prices: subsistence output increases 0.7%. Commercial agriculture contracts further: its output and surpluses decline 25% and 46%, respectively, while purchases from outside the locality continue to surge (Table 2, d).

Since carbon prices are fixed throughout the program’s expansion (i.e., the unit cost of carbon services is constant), public costs and benefits both double. Expanding the program also entails a 112% increase in net private costs, which now amount to $103,300 in real terms (Fig. 3). Subsistence demands still prevent many households from joining the program, and the income effect similarly reduces the amount of land that participants are willing to commit. Some economic rents are available to participants at first, but these diminish as the program expands and market rents rise (Fig. 4d). Thus, the expansion reduces participants’ incentives by raising their opportunity costs. Inefficiencies decrease, but enrollment in PES becomes a liability when market rents rise above program payments. This occurs when >18% of land is enrolled. A marked redistribution of gains and losses follows.

Expanding the program helps transfer participants’ gains to landowners in general, redistributing the gross benefits of carbon management more equitably; but the outcome still is unfavorable for most stakeholders (Fig. 4e). Households’ terms of trade are highly disadvantageous, and economic rents are insufficient to generate net benefits or even offset wage-income losses. Incomes decrease by 1.7% for both local households in general and the average participant. Only landlords draw net benefits from the expansion given that their own terms of trade improve, but their gains are redistributed (Fig. 4f). Unable to devote more land to carbon sequestration, local landlords depend on economic rents for a gain. As the program expands (and economic rents fall), their benefits diminish, ultimately becoming losses ($2,500) when the area covered doubles. These agents in fact might consider opting out of the program, for it is now more profitable to rent land to farmers. In contrast, absentee landlords continue enrolling land as the program expands and market rates rise. Their gains grow steadily because land rents, not economic rents, constitute their main source of revenue. Their income increases by 15% or $8,700.

Overall, the changes implemented have a mixed outcome. In terms of efficiency, the program now generates twice as many public benefits as in scenario 1a but only 52% more costs. In terms of equity, reforms reduce inequalities between participants and non-participants but fail to improve local livelihoods. Changes mostly transfer the benefits from the largest participants to the largest landowners (i.e., from local to absentee landlords) and the costs from the general public to local households. Efficiency is restored at the expense of local stakeholders. Avoiding economic rents completely requires removing price controls and constraints on participation simultaneously, that is, achieving the program’s enrollment target via supply-and-demand equilibrium. Scenario 1c contemplates program changes along these lines, revealing the costs and benefits of expanding a cost-effective program (see Supporting Information).

All scenarios up to this point are based on the assumption that wages are flexible. Since wages tend to decrease, this assumption allows farmers in the model to increase their use of labor. The assumption is justified only if labor markets are small or relatively isolated, e.g., when workers have few short-term opportunities outside the farm. Our final scenario relaxes the flexible-wage assumption to analyze the case when wages are unchanged after an intervention.

#### Scenario 2. A cost-effective program in a fixed-wage context

We begin this last scenario by simulating a program that offers competitive prices to enroll 10% of arable land, as in the first scenario, except that opportunities for off-farm employment are now widely available (i.e., labor demand is perfectly elastic), which keeps wages constant. Then we expand enrollment from 10 to up to 20% of land (in what constitutes the fixed-wage equivalent of scenario 1c).

Constant wages have two opposite effects on both local demand for land and the opportunity costs of enrollment that have implications for carbon prices and program participation. First, constant wages discourage the use of farm labor (relative to previous scenarios), decreasing the intensity of labor (per unit area) and hence the productivity of land. The lower returns discourage demand for arable land and reduce the opportunity cost of enrolling in PES. In such circumstances, program administrators can offer prices only 3.7% higher than market rental rates (compared to 7% in the first scenario) and still reach its target (Table 2, col. e). The implication is that the incentives (or economic rents) necessary to induce participation are nearly half those required with a flexible wage. At the same time, constant wages help sustain household incomes, thus maintaining subsistence demands relatively intact. Wages reflect labor productivity, which is sustained by lowering the intensity of on-farm labor. Laid-off workers find employment off-farm nevertheless, and workers in general face better terms of trade than in previous scenarios. However, farmers’ reluctance to substitute labor for land (due to the high wage) constrains subsistence output, which becomes a scarce commodity–i.e., the program raises its implicit value. This raises the returns to and demand for land (compared to the first scenario), raising the opportunity costs of enrolling in PES. The implication is that only 21% of households participate in the program and contribute 84% of its target (compared to 40% and 97%, respectively, in scenario 1) (Table 2). Absentee landlords now enroll six times more land than in a variable-wage economy.

Constant wages also have important implications on the local economy, particularly agricultural output, which declines by 8.3% or almost thrice as much as in scenario 1 (Table 2). Subsistence output declines for the first time but still marginally (0.3%) vis-à-vis commercial output, which contracts 28% (compared to 12% in the first scenario). Market surpluses decrease 52% as a result (down from 22%), and the local deficit increases by a record 9.3% (up from 2.9%). Absentee and local landlords’ income increases 3.7 and 0.5%, respectively, or $2,200 and $1,100. This represents a slight improvement for local landlords (compared to the first scenario) but a relative loss of $1,850 for absentee landlords (due to relatively lower rents). In contrast, the average household experiences no change in income–a relative gain with respect to the flexible-wage scenario. In fact, no household experiences losses now. Average gains for participants are 0.1% in real terms; non-participants experience no visible change. Since there are no economic rents and wages are constant, the small differences between these groups are due to the weight of land in their asset portfolio; i.e., in the fixed-wage scenario, costs and benefits are distributed exclusively through land rents.

In sum, constant wages diminish the productivity of land while increasing the value of subsistence output. The effect of these opposing forces on the returns to land–which ultimately determine the opportunity cost of enrollment into PES, the price of carbon and the distribution of gains and losses–is an empirical question. In our model economy, constant wages have a much stronger effect on production than consumption, and so rents are considerably lower than when wages are flexible. Constant wages thus reduce the private costs of PES more than its benefits and redistribute both more equitably. Significant net private losses (in scenario 1) now become net gains of $2,900 in real terms (Fig. 3). Constant wages also reduce the program’s public costs to $225,300 (given that carbon prices are lower) or 3% less than when wages are flexible. And since no household experiences losses, there are no private costs to compensate. The bottom line is a 21% decrease in the social costs of carbon services relative to the first scenario.

The previous estimate suggests that if off-farm employment opportunities were few (as in scenario 1), the program could employ laid-off farm workers (instead of compensating them for their losses) and still reduce its public costs by 6% (Fig. 3). By becoming an employer of last resort, program administrators prevent the wage rate from dropping, which keeps labor costs high but simultaneously protects workers’ earnings and improves households’ terms of trade. This combination of effects mitigates the excess demand for land induced by PES, reducing the intervention’s costs to all stakeholders. A “working” program thus achieves greater efficiency and equity without compromising its own effectiveness. The critical question is whether these results are independent of program size.

Simulation results show that both production and consumption effects persist when the program covers 20% of the land, but their relative weights change (Table 2, col. f). The effect of labor costs on production is constant throughout the expansion–i.e., depressing the marginal productivity of land–while labor earnings’ effects on consumption increase. Incomes grow slowly, but arable land becomes scarce very rapidly and the implicit value of subsistence output rises noticeably, discouraging enrollment into PES. Although carbon prices are low at first (cf. scenario 1c), they must increase faster than when wages are flexible in order to achieve the expanding target (Fig. 3g). An increasing number of landowners join the program–i.e., up to 52% of households–given that the opportunity cost of farming rises rapidly, but land enrollment does not increase at the same rate: when the target is doubled, local landowners contribute 59% of the total (compared to 62% in scenario 1c) (Table 2). Moreover, doubling enrollment requires a payment 17% above the original market rents, which implies higher carbon prices than under flexible wages.

Other economic repercussions also are significant. Agriculture contracts up to 10%, commercial output and market surpluses decline up to 32 and 57%, respectively, and food purchases in the open market increase up to 10%–the greatest recorded. The distribution of costs and benefits also differs markedly from flexible-wage scenarios (Fig. 4h). While wage income remains unchanged, changes in rental incomes (or outlays) have no significant influence on the average household (who experiences losses in a flexible-wage economy). Moreover, all program participants now experience marginal gains, and only 4% of non-participants experience marginal real-term losses. Nevertheless, significant gains are available only to large landowners: local and absentee landlords gain up to 2.0 and 17% more, respectively, or $5,000 and $9,700 (Fig. 4i).

Expanding the program requires substantial resources, raising the public costs to $507,100–or 1% more than under flexible wages (Fig. 3). Providing employment to laid-off farm workers adds $40,333 to these costs; but since there are no additional losses to compensate, the social costs remain 10% lower than under the next-best alternative (see Supporting Information). The program also generates net private gains equal to $41,500 in nominal terms, but given the rising cost of subsistence consumption, this represents real term gains of only $14,400 (Table 2). Overall, a “working” program remains the most efficient and equitable option within the range examined.

### Generality of Results

To assess whether the study area is representative of other areas in Mexico, we focus on two conditions crucial to our theory of change: 1) the distribution of productive assets among local households and absentee landowners; and 2) the persistent allocation of these assets to subsistence agriculture. That is, the question is whether we can justify modeling an “income effect” on subsistence production but not an analogous response from commercial producers facing credit restrictions.

Landholding sizes vary considerably throughout Mexico, with large farms often interspersing with more numerous smallholdings [32]. In almost every region, the rental market helps redistribute land among rural farmers, and absentee landowners supply up to 88% of these markets [33]. The figure in Zoatecpan, 80%, is representative of other localities in central Mexico, 83%. Although most land in Zoatecpan already is sown in maize, farmers respond readily to incentives by renting land in or out [9]. Subsistence farmers expand maize production when their income increases but not in response to higher prices, while commercial farmers respond the opposite way [34]. Similarly, across rain-fed areas in Mexico, commercial farmers do not invest exogenous income on maize production [35]. Zoatecpan is outside the norm as regards subsistence production nevertheless. Across Mexico, rural farmers consumed an estimated 48% of their maize output in 2002 [34]. On-farm consumption was highest in central Mexico, reaching 73%, while the figure in Zoatecpan was 85%–a reflection of small landholding sizes there. In sum, while not representative of prime agricultural areas in Mexico, our results should apply across the densely-populated, highly-deforested tropical highlands. More generally, we should expect a similar outcome to that described here wherever subsistence agriculture is widespread, population density high and landholdings small.

## Discussion

### REDD+ Policy Options

If efficiency is a goal for REDD+, market-based mechanisms are probably the best alternative [4], [36]; but unless REDD+ becomes part of a common carbon market, program administrators would not necessarily be price takers. Certainly, authorities might be forced to pay competitive rates for land in order to achieve precise targets in a locality, but they could relinquish these targets in exchange for the capacity to decide the price paid. This price nevertheless may not be the rental rate prevailing at present in each locality. In the study area, for example, absentee landowners presumably are willing to set aside up to 30% of the total land area at such rate, assuming no long-term commitments are involved. They already rent this land out to local farmers and are indifferent between alternative tenants or land uses; i.e., for them, the opportunity cost of enrolling land in PES is the current market rate (and their supply of land at this rate is perfectly elastic). However, their response to a full-scale REDD+ scheme could alter land markets significantly, and this would reshape their own decisions in turn. Overall, the opportunity cost of land for these actors could increase (i.e., and the supply of land become more inelastic) as REDD+ unfurls, contrary to the assumptions of studies where opportunity costs remain constant unless agricultural prices change [10]–[12], [14].

Figure 4g illustrates this point. Since carbon prices in scenarios 1c and 2 are equivalent to rental rates, the lines describing the evolution of rents as a function of program area also describe carbon prices; i.e., these lines represent the study area’s supply of carbon services (its supply curve) under two slightly different sets of conditions. The graph shows that supply is not exceptionally responsive to prices (i.e., it is relatively inelastic). Authorities must pay 7% more than baseline rates to enroll 10% of the land, but enrolling 20% requires 17% more. This is not because opportunity costs differ across the landscape and less-productive land is enrolled first, as in previous simulation studies, since we assume a common market for land of uniform quality. Opportunity costs increase because of REDD+. The reason is that subsistence farmers will pay higher rents for land as subsistence goods and services become scarce and their value increases. Also evident is that enrollment into PES (i.e., the slope and intercept of the supply curve) depends on economic conditions beyond the land market, particularly those involving labor. More generally, the opportunity costs of alternative land uses, including enrollment into PES, are not a function of biophysical factors and international commodity prices alone, as often assumed [10]–[12], [14], [37]. In a subsistence economy, opportunity costs are influenced by consumption preferences expressed in a particular social, cultural and market context.

Assessing the 3Es of alternative REDD+ programs requires accounting for this context. At the aggregate level, there is a clear trade-off between the efficiency and equity of PES: as carbon prices rise, a program’s private gains increase in direct proportion to its public costs. However, this relationship blurs when the distribution of costs and benefits is considered. In our model economy, raising carbon prices creates substantial benefits for some participants, but it does not compensate most agents’ losses (Fig. 4b). As discussed earlier, benefits do not trickle down because the system is considerably open (and the multiplier effect of cash injections is small): as soon as cash flows in via PES, it is siphoned out through markets for consumer goods, which are more tightly integrated across the country than land or labor markets. On the whole, raising carbon prices has a toll on the program’s efficiency while failing to solve its inequities. Expanding the program reduces the inefficiencies while distributing private costs more evenly, but these costs also increase substantially. To keep both public and private costs under control while preserving the program’s effectiveness, authorities would need to intervene in labor markets to prevent job losses.

Given the difficulty of balancing these multiple goals, it should not surprise that the 3Es of PES programs in Mexico have been called into question [38], [39]. Simulations reveal that, at best, REDD+ could have a negligible impact on households in the study area; but the robustness and generality of our findings, as those of any simulation exercise, rest on the validity of underlying assumptions [15]. Scenario 2 highlights the sensitivity of our results to closure rules, which reflect different assumptions on market conditions; and figure 4 (top row) illustrates how these conditions determine the value of agents’ assets. Namely, the two program characteristics examined here–carbon prices and program area–have similar qualitative effects on value added when off-farm employment is scarce, they raise rental rates while depressing wages (Fig. 4a, d); but value added is redistributed exclusively through changes in rental rates when employment opportunities are widely available (Fig. 4g). These two characteristics also have contrasting effects on economic rents regardless of employment opportunities in the area: increasing prices generate these rents, the program’s expansion dissipates them (Fig. 4a, d). A robust conclusion thus requires recognizing that individual program characteristics, the context in which the program is implemented, and the interaction of program design and context all play a decisive role [17].

Our theory of change posits that it is ultimately these three sources of value–wages, rental rates and economic rents–that determine the incentives and social outcome of the various alternatives (Fig. 4, middle and bottom rows). Thus, authorities’ ability to address program shortcomings is restricted by the shape of rental and wage-rate functions (Fig. 4, top row). Alternative specifications for demand and supply of land and labor could result in non-linear wage and rent functions (as illustrated in Fig. 1). Yet, there is little empirical evidence to support other than the simple (and arguably most neutral) specification adopted here (see Supporting Information) [40]. This modeling decision reflects the current state of knowledge of rural economies in Mexico and throughout the developing world.

### Conclusions

Is there an ideal REDD+ program that is effective, efficient and equitable? Our results show that when the diversity of local actors is taken into account, the relationship between the 3Es is complex and characterized by numerous trade-offs. Choosing a second-best alternative requires normative judgments and a precise knowledge of the trade-offs involved. A program that minimizes its public costs could entail significant private losses. Improving the program’s equity while preserving its effectiveness would require transforming those losses into public costs. Whatever mechanism is adopted at the international level, it should avoid general formulas by giving local authorities the necessary flexibility–e.g. in terms of scope, form and amount of compensation–to address these trade-offs. The best option will depend on local conditions, so national programs themselves should remain flexible enough to adjust for spatially and temporally changing contexts.

Setting land aside as a carbon sink entails changes in the productivity of land and, whenever subsistence households are present, the value of agricultural output. Its effects on the returns to land could determine the opportunity cost of enrolling land in REDD+ and therefore influence carbon prices, program areas and the distribution of gains and losses. However, given that these effects have opposite signs, their outcome will change from place to place. In the tropical highlands of Mexico, expanding a program or raising the price paid for carbon services could fail to benefit most rural households, particularly subsistence households or those whose main asset is labor. A program that prevents job losses might be the best option, but its efficiency–e.g., compared to direct compensation–could depend on the program’s scale and the extent of demand for agriculture’s non-market benefits in the locality.

Our conclusions apply mainly to the densely-populated highlands, but the processes described could occur more widely. Ideally, then, REDD+ should be conceived as a place-based policy, but this assumes that tailoring programs to local conditions will not result in a loss of accountability. Also, authorities would need sufficient knowledge of these conditions to identify the most suitable option. Our methodological framework can be applied at much wider scales provided adequately representative data is available or collected [33]. It is a reliable tool for the design of both REDD+ programs and the integral rural development policy of which REDD+ should become part.

## Supporting Information

Supporting Information S1 (DOC)
