# Supplementary material for: Is There an Ideal REDD+ Program? An Analysis of Policy Trade-Offs at the Local Level
Source: PLoS One. 2012 Dec 26;7(12):e52478. doi: 10.1371/journal.pone.0052478 (PMC3530448; doi:10.1371/journal.pone.0052478)
Supplement: Supporting Information S1 — (DOC) [file pone.0052478.s001.doc]

**Supporting Information**

***Model.*** Our modeling framework integrates 49 individual farm-household models into a general equilibrium model of a village economy. Consumption preferences and production technologies are of the same general form for all households, but they are parameterized distinctly so that production and consumption decisions reflect each household’s preferences and technology. Given that households are heterogeneous producers and consumers, they interact with each other in local markets. This sets the model apart from conventional CGE models in which production is undertaken by a representative agent and consumption is aggregated into household types. It also represents the heterogeneous rural economy of developing areas more realistically. In many of these areas, commercial producers of staple foods coexist with subsistence farmers that produce a composite agricultural good that often includes the same tradable staples produced by commercial farmers as well as associated non-tradable goods or services [1]. Subsistence agriculture thus is guided by endogenous shadow prices that can be considerably higher than the market price of its tradable output [1, 2].

Each household in the model faces the following optimization problem:

where *U* is a standard, quasi-concave utility function; *A* represents consumption of the composite agricultural good; *C = (C_1_, C_2_,…,C_I_)* represents consumption of *I* tradable goods of which the first *m* goods are produced by the household; *Z* is leisure, and *β* is a vector of the household’s preference parameters. Equation 1 is the household’s cash-income constraint, where *p_i_* and *Q_i_* are the price and output of good *i*; *Q_A_* and *p_A_* are agricultural output and prices; *w* and *r* are local wage and land rental rates; *L_A_* and *D_A_* are the amount of labor and land used in agriculture; *L_i_* and *D_i_* are labor and land used in the production of good *i*; $\bar{L}$ and $\bar{D}$are the household’s labor supply and land endowment; and $\bar{Y}$is net exogenous income from remittances and government transfers. Equation 2 gives the household’s technology constraints, where the output of each good is assumed to exhibit constant returns in labor, land, and capital, *k* (which is fixed), given the household’s production technology *γ*. Equation 3 is the household’s time constraint, where *X* is commuter work outside the locality and $\bar{T}$ its time endowment. An additional constraint, equation 4, restricts consumption of the composite agricultural good to the household’s output. This constraint is binding for subsistence households, making their optimization problem not recursive [3]. Subsistence households can buy staple foods at a market price, but the price of the composite good is given by the household’s endogenous shadow price, $\rho_{A}^{h}$ > *p_A_* [4]. Commercial-farm households are defined by a non-binding subsistence constraint. They might produce and consume agricultural non-market goods and services, but their marginal value is nil for these households; so the price of agricultural output, *p_A_*, is equal to the market price for agricultural tradables. Therefore, these households maximize profits, and their optimization problem is recursive. Profit maximization, in turn, implies that demand for land and labor is a function only of the household’s capital endowment, its specific technology parameters, market prices, wages and rents. Utility maximization subject to the full-income constraint yields commercial households’ consumption demand. Under the assumption of non-joint production and some fixity of capital, subsistence households behave as profit maximizers with respect to non-agricultural goods that they produce. Thus their factor demands in the production of these goods have the same form as those of commercial households. However, their factor demands in agriculture as well as their consumption demands are functions of household preferences.

In a second stage, the individual household models are linked together into a village-wide general equilibrium model. Equations 5 and 6 represent village-wide constraints equating total demand for land and labor minus total endowments to net demand for land, *D^v^*, and labor, *L^v^*, from neighboring villages. Equation 7 equates net demand for food to the village’s net surplus, *Q^v^*. Trade balances are fixed in scenarios where land, labor and food markets are closed, yielding endogenous (i.e., village) factor and food prices.

The model was calibrated with a disaggregated social accounting matrix (SAM) and solved using GAMS software.

***Scenario 1c. Expanding a cost-effective program.*** In the first scenario program administrators enroll 10% of land at a minimum cost by offering competitive prices, precluding economic rents. In the present scenario administrators offer increasingly higher prices to enroll up to 20% of the land under the assumption that wages are flexible. In our model economy, expanding the program two fold requires a payment 15% higher than the original market rents (Table 2, col. g). This is a percentage point above the price paid in the scenario 1b for the same amount of land; but prices can be up to 6% lower when the target is lower. That local participation and enrollment do not differ significantly from the previous scenario is not surprising, considering the definition of economic rents. Changes in rents and wages and their repercussions also are basically the same (as in scenario 1b’s constant-price expansion) (Table 2).

Economic rents might have few multiplier and general-equilibrium effects, but eliminating these rents by offering competitive prices has a considerable impact on the distribution of costs and benefits. Since paying market prices is efficient, the program’s private costs increase at a lower rate than its private benefits (i.e., 108 and 123%, respectively); but absolute costs are still greater than absolute benefits (i.e., $139,600 and $9,200), and net private losses grow to $99,500 in real terms. In contrast, the program’s expansion could entail a net public gain even when its public costs increase at a higher rate than its public benefits—i.e., 115 and 100%, respectively. Market prices also redistribute gains and losses among private stakeholders. Since economic rents are absent, program payments become the opportunity cost to every farmer (i.e., the graph in Fig. 3g represents land rents and carbon prices). This means that in contrast to scenarios 1a and 1b, where limits to enrollment and price controls grant participants exclusive access to program benefits, participants now experience exactly the same costs and benefits as non-participants, *ceteris paribus* (Fig. 3h). As before, this does not imply that there are no differences between other groups, e.g., landowners and working families. Simultaneous increases in carbon prices and enrollment area raise absentee landlords’ income by 15% or $8,900 (Fig. 3i). At least one aspect of subsistence households’ wellbeing also improves: on-farm consumption is greater than when only prices or enrollment are raised (i.e., scenarios 1a and 1b). But this still fails to generate a net gain for the average household: 4% of households experience marginal income gains, while the rest is worse off than without the program. In fact, average real-income losses increase from 0.8 to 1.6% as the program expands. Again, the reason is that households’ terms of trade become highly disadvantageous due to wage decreases.

In sum, the program’s expansion via competitive carbon prices is efficient, but it exacerbates its inequity. In order for the program to be Pareto optimal, program administrators need to compensate individual losses amounting to $109,300, raising total costs by 22%. These costs are 11% higher than those incurred in scenario 2’s “working” program.

1. Smale M, editor (2005) Valuing crop biodiversity: On-farm genetic resources and economic change. International Plant Genetic Resources Institute (IPGRI), CABI Publishing

2. Arslan A, Taylor JE (2009) Farmers’ subjective valuation of subsistence crops: the case of traditional maize in Mexico. Am J Agric Econ 91: 956-972.

3. Dyer G, Taylor JE, Boucher S (2006) Subsistence response to market shocks. Am J Agric Econ 88: 279-291.

4. De Janvry A, Fafchamps M, Sadoulet E (1991) Peasant household behavior with missing markets: Some paradoxes explained. Econ J 101:1400-1417.
